# I see the light! Fluorescent proteins suitable for cell wall/apoplast targeting in *Nicotiana benthamiana* leaves

**DOI:** 10.1002/pld3.112

**Published:** 2019-01-17

**Authors:** Angela Stoddard, Vivien Rolland

**Affiliations:** ^1^ CSIRO Agriculture & Food Canberra Australian Capital Territory Australia

**Keywords:** *Agrobacterium tumefaciens*, agroinfiltration, apoplast, AT5G11420, fluorescence, fluorescent proteins, Gamillus, mCherry, mCitrine, mEYFP, mNeonGreen, mTurquoise2, *Nicotiana benthamiana*, pH, pH‐tdGFP, plant cell wall, RPP3A, sfGFP, TagRFP, transient expression

## Abstract

Correct subcellular targeting is crucial for protein function. Protein location can be visualized in vivo by fusion to a fluorescent protein (FP). Nevertheless, despite intense engineering efforts, most FPs are dim or completely quenched at low pH (<6). This is particularly problematic for the study of proteins targeted to acidic compartments such as vacuoles (pH ~ 3–6) or plant cell walls (pH ~ 3.5–8.3). Plant cell walls play important roles (e.g. structural/protective role, control of growth/morphogenesis), are diverse in structure and function, and are highly dynamic (e.g. during cell growth, in response to biotic/abiotic stresses). To study and engineer plant cell walls, it is therefore critical to identify robust tools which can be used to locate proteins expressed in the apoplast. Here we used a transient expression assay in *Nicotiana benthamiana* leaves to test a range of FPs in vivo*,* and determined which ones retained strong fluorescence in the acidic environment of the apoplast. We selected 10 fluorescent proteins with a range of in vitro properties; two historical FPs and eight FPs with in vitro properties suggesting lower pH sensitivity or improved brightness, some of which had never been tested in plants prior to our study. We targeted each FP to the cytosol or the apoplast and compared the fluorescence in both compartments, before testing the in vivo pH sensitivity of FPs across a pH 8–4 gradient. Our results suggest that mTurquoise2, mNeonGreen, and mCherry are suited to tracking proteins in the apoplast under dynamic pH conditions. These fluorescent proteins may also be useful in other acidic compartments such as vacuoles.

## Short Abstract

In vivo comparison of fluorescent proteins with various degrees of in vitro sensitivity to low pH identifies tags suitable for tracking proteins in cell walls under dynamic conditions.

## 1 INTRODUCTION

Endogenous and foreign proteins must be targeted to a particular cellular or extracellular location in order to carry out their function. In vivo tracking of a protein of interest can be achieved by its fusion to a fluorescent protein (FP), which can absorb and emit light of specific wavelengths. The first green FP (*Av*GFP) was isolated from the *Aequorea victoria* jellyfish, while the first red FP (*Ds*Red) was cloned from a *Discosoma* coral species (Matz et al., 1999; Prasher, Eckenrode, Ward, Prendergast, & Cormier, 1992; Shimomura, Johnson, & Saiga, 1962).

Some of the properties of wild‐type FPs limit their suitability as markers in transgenic organisms. For example, their absorption spectra are complex and broad, they function as multimers, and their optimal maturation temperature is well below that of mammalian cells (Baird, Zacharias, & Tsien, 2000; Chalfie, Tu, Euskirchen, Ward, & Prasher, 1994; Matz et al., 1999; Siemering, Golbik, Sever, & Haseloff, 1996). To circumvent these shortcomings, considerable effort has focused on tailoring the properties of these FPs [e.g. protein folding, maturation, brightness, specificity of light absorption/emission, monomerization, pH stability, photo‐stability, and thermo‐stability; for reviews, see (Day & Davidson, 2009; Shaner, Steinbach, & Tsien, 2005)]. However, the intrinsic properties of FPs are interconnected and thus the improvement of a particular attribute often comes at the expense of other characteristics.

Imaging plants tissues poses unique challenges because of their autofluorescent compounds (e.g. chlorophyll, flavonoids, alkaloids, tannins) and structures (e.g. chloroplasts, cell walls, cuticle) (García‐Plazaola et al., 2015). Visualizing FPs in the apoplast (i.e. cell walls and intercellular spaces) is particularly challenging because in addition to autofluorescence, the pH of the apoplast is often acidic (e.g. 5.4 in meristematic cells of Arabidopsis roots) and can vary widely (pH 3.5–8.3; e.g. depending on the tissue/species, if cells are growing, or if they are responding to biotic or abiotic stresses) (Arsuffi & Braybrook, 2018; Barbez, Dunser, Gaidora, Lendl, & Busch, 2017; Geilfus, 2017; Yu, Tang, & Kuo, 2000). This limits the functionality of FPs in the apoplast because acidic conditions reduce FP fluorescence [for an *in planta* example, see Dean et al. (2007)]. The sensitivity of a FP to low pH is captured in vitro by its p*K*a: the pH value at which 50% of the protein pool can fluoresce. In theory, the lower the p*K*a, the more stable the FP fluorescence at low pH. The p*K*a of some of the most commonly used FPs (e.g. EGFP – p*K*a_EGFP_ = 6.0, sfGFP – p*K*a_sfGFP_ = 5.9, EYFP – p*K*a_EYFP_ = 6.9) suggests that they are less than ideal to track proteins in acidic environments (Miyawaki, Griesbeck, Heim, & Tsien, 1999; Roberts et al., 2016; Sarkisyan et al., 2015). Moreover, p*K*a measurements are performed in vitro and may not reflect *in planta* protein behavior. Other protein characteristics, such as brightness, stability, and spectral overlap with tissue autofluorescence, are likely to play a critical role in vivo. FPs with promising in vitro properties must therefore be tested in vivo to identify tags best suited to a given cellular or extracellular compartment. While certain fluorescent proteins have been used in the apoplast (e.g. see Albenne, Canut, Hoffmann, and Jamet (2014)), their in vivo ability to withstand rapid pH changes has not been tested.

In this study, we compared the pH‐sensitivity of different FPs by using a series of 10 FPs predicted to have improved pH stability or brightness, along with some of the ancestral proteins they are derived from (Table 1). Each fluorescent protein was targeted to the cytosol or the apoplast by fusion to the C‐terminus of *Arabidopsis thaliana* RPP3A (a small cytosolic protein of 119 amino acids) or AT5G11420 (an uncharacterized cell wall protein of 366 amino acids), respectively, and the fluorescence in the two compartments was compared (Albenne et al., 2014; Cutler, Ehrhardt, Griffitts, & Somerville, 2000). We also investigated their in vivo fluorescence across a pH gradient, to identify proteins with reduced pH sensitivity which may assist cell‐wall engineering efforts and allow protein tracking in other acidic environments, such as vacuoles.

**Table 1:**
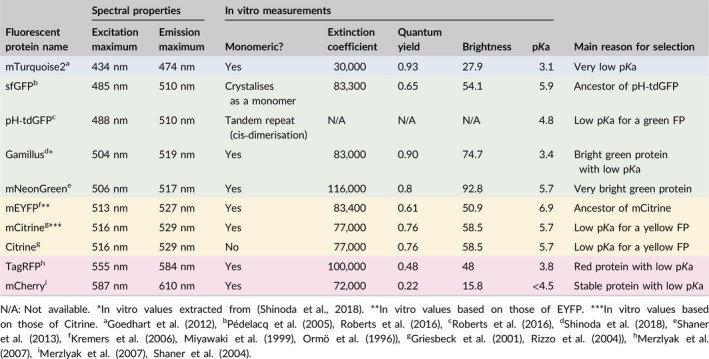
Main properties of the fluorescent proteins tested in this study. Most of this data is available at http://www.fpvis.org/FP.html. The extinction coefficient (M^−1^ cm^−1^) is a measure of how well a FP absorbs light while the quantum yield is a measure of how well a FP emits light. Brightness is the product of the extinction coefficient and the quantum yield, divided by 1000. The color of a row indicates the color range of the light emitted by the fluorescent protein

## 2 MATERIALS AND METHODS

### 2.1 Protein alignments

Protein alignments were generated using ClustalW in Geneious R10.2.2^®^.

### 2.2 Cloning of the constructs

The gateway vectors pMDC‐mEYFP, pMDC‐Citrine, pMDC‐mCitrine, pMDC‐sfGFP, pMDC‐pH‐tdGFP, pMDC‐mCherry, pMDC‐Gamillus, pMDC‐mNeonGreen, pMDC‐TagRFP, and pMDC‐mTurquoise2 were generated in this study. Briefly, each pMDC‐FP vector was created by replacing the 184 bp SalI/SacI fragment of pMDC32 (Curtis & Grossniklaus, 2003) with a fragment containing a SalI site, an attR2, and a flexible linker sequence (fragment sequence: GTCGACCATAGTGACTGGATATGTTGTGTTTTACAGTATTATGTAGTCTGTTTTTTATGCAAAATCTAATTTAATATATTGATATTTATATCATTTTACGTTTCTCGTTCAGCTTTCTTGTACAAAGTGGTTCGATTCCTTAATCTAGTTCTAGAGCGGCCGGGCGGTGGCAGC) placed at the 5′ of each FP, which was followed by a SacI site. A 4xGly motif was introduced before the stop codon of each FP to inhibit the possible mis‐targeting effect of their C‐terminal MDEL motif, as previously described (Nishizawa et al., 2003). SalI/SacI fragments were synthesized by Genewiz (New Jersey, USA) or Geneart (Massachusetts, USA).

All plant expression vectors containing RPP3A‐FP and AT5G11420‐FP fusions were generated by recombining entry vectors containing the coding sequence of *Arabidopsis thaliana* RPP3A (AT4G25890.1) or AT5G11420 (AT5G11420.1), respectively, with each pMDC‐FP (promoter: 2 × 35S, resistance: Kanamycin) following standard LR gateway reactions. Entry vectors contained attL1‐RPP3A‐attL2 and attL1‐AT5G11420‐attL2 sequences in which the stop codons had been removed to allow in‐frame fusion with FPs. These sequences were synthesized by Genewiz (New Jersey, USA).

### 2.3 Plant material and growth conditions

*Nicotiana benthamiana* plants were grown for 5 weeks in a CONVIRON growth chamber with 16 h/8 h day/night at 23°C and approximately 100 μmol photons m^−2^ s^−1^ light intensity.

### 2.4 Agroinfiltration of *Nicotiana benthamiana* leaves

Agroinfiltration was carried out essentially as described in Rolland (2018). In short, *Agrobacterium tumefaciens* GV3101(pMP90) (Koncz & Schell, 1986) was transformed with plasmids of interest (POIs) and grown in LB media containing rifampicin (50 μg/ml) and kanamycin (25 μg/ml). Cultures were grown for about 24 h in a 28°C incubator and used for transformation of *N. benthamiana* leaves. A vector encoding the tomato bushy stunt virus P19 protein was used to inhibit post‐transcriptional gene silencing and to allow other constructs to be expressed (Roth, Pruss, & Vance, 2004). For each infiltration, a volume of P19‐containing bacteria corresponding to OD_600_ = 0.3 was mixed with a volume of POI‐containing bacteria corresponding to OD_600_ = 0.5. Cells carrying P19 alone were used as a negative control. Cells were centrifuged for 8 min at 2150 ***g*** and resuspended in a solution of 10 mM MES pH 5.6, 10 mM MgCl_2_, and 150 μM acetosyringone. The cells were incubated for 2 h at room temperature and infiltrated into the abaxial side of 5‐week old *N. benthamiana* leaves.

### 2.5 Preparation of leaf samples and confocal laser‐scanning microscopy

In all experiments, fresh leaf disks of 2.2 cm in diameter were harvested 3 days post infiltration (dpi) from leaves transformed with P19 alone, P19 + RPP3A‐FP, or P19 + AT5G11420‐FP fusions. The time point of 3 dpi was selected because in our hands there is no detectable protein expression at 1 dpi, and at 5 dpi expression levels tend to be too high and proteins start to accumulate in other compartments. These disks were imaged with a Leica SP8 confocal laser‐scanning microscope (Leica Microsystems, Australia) equipped with a 40× (NA = 1.1, for cytoplasm/cell wall localization and plasmolysis experiments) or a 10× (NA = 0.3, for pH series experiments) water immersion objective. Images were acquired with the Leica LASX software.

To assess protein localization in abaxial epidermal cells, fresh leaf disks were mounted in water and imaged immediately. In plasmolysis experiments, leaves were infiltrated with either 30% (v/v) glycerol (plasmolyzed condition) or water (unplasmolyzed control), using a 1 ml syringe, and fresh disks were harvested immediately for imaging.

The sensitivity of FPs to pH was tested in the spongy mesophyll tissue to ensure that the apoplast could be efficiently immersed in a buffered solution, which would have been slower and possibly inconsistent in the epidermal layer with its thick waxy cuticle. More specifically, a small circle was delicately drawn on the adaxial side of each disk transformed with the fluorescent construct of interest. This ensured that the same group of cells was observed throughout the experiment. The abaxial epidermis of each disk was then peeled off using forceps to expose the spongy mesophyll. Peeled disks were then mounted in 50 mM HEPES pH 8, and the cells within the circle were imaged immediately. Imaging settings were adjusted to avoid detector saturation as well as to limit background tissue fluorescence (as observed in P19 negative controls imaged with the same settings). The solution was then replaced with 50 mM PIPES pH 7, and the same cells were imaged with identical settings. This process was repeated on the same disk with solutions of 50 mM MES pH 6, 50 mM Sodium acetate pH 5, and 50 mM Sodium acetate pH 4. After imaging at pH 4, the disk was placed in 50 mM HEPES pH 8, to assess whether fluorescence could be restored.

### 2.6 Fluorescence quantification across a pH 8 to pH 4 series

For each construct, the fluorescence emitted by the same group of cells across a pH 8–4 gradient was quantified as follows, using FIJI and Image J 1.52 h (Schindelin et al., 2012; Schneider, Rasband, & Eliceiri, 2012). To account for slight tissue rotation induced by the repeated replacement of buffered solutions, the cumulative fluorescence value (called RawIntDen in Image J) of a disk 512 μm in diameter was measured in each image. The chlorophyll channel (not shown) was used to ensure that disks were placed over the same group of cells across a pH series (see Figure 5a‐a'). Background fluorescence (as measured in a disk of 512 μm in diameter placed over P19‐expressing cells immersed in 50 mM HEPES pH 8, and imaged with identical settings) was deducted from each measurement in a given pH series. Fluorescence intensities were then normalized to the fluorescence measured at pH 8 (arbitrarily set at 100%). Three independent pH series were quantified per construct.

### 2.7 Confocal laser‐scanning microscope settings

**Table:** 

Protein name	Sequential scanning	λ_ex_	λ_em_ (fluorescent protein)	λ_em_ (chloroplasts)	Transmitted light	Notch filter
mTurquoise2	No	458 nm	470–495 nm	650–690 nm	Yes	458/514 nm
sfGFP	No	488 nm	500–520 nm	650–690 nm	Yes	488 nm
pH‐tdGFP	No	488 nm	500–520 nm	660–680 nm	Yes	488 nm
Gamillus	Sequence 1	504 nm	510–530 nm*	No	Yes	488/561/633 nm
	Sequence 2	633 nm	No	650–690 nm	No	
mNeonGreen	No	506 nm	512–530 nm*	650–690 nm	Yes	None
mEYFP/Citrine/mCitrine	No	514 nm	520–540 nm	650–690 nm	Yes	514 nm
TagRFP	Sequence 1	555 nm	570–600 nm*	No	Yes	488/561/633 nm
	Sequence 2	633 nm	No	650–690 nm	No	
mCherry	Sequence 1	587 nm	610–630 nm	No	Yes	488/561/633 nm
	Sequence 2	633 nm	No	650–690 nm	No	

* In the pH series experiments, these detectors were gated (0.3–6 ns) to reduce background fluorescence.

## 3 RESULTS

### 3.1 Properties of selected fluorescent proteins

**Figure 1:**
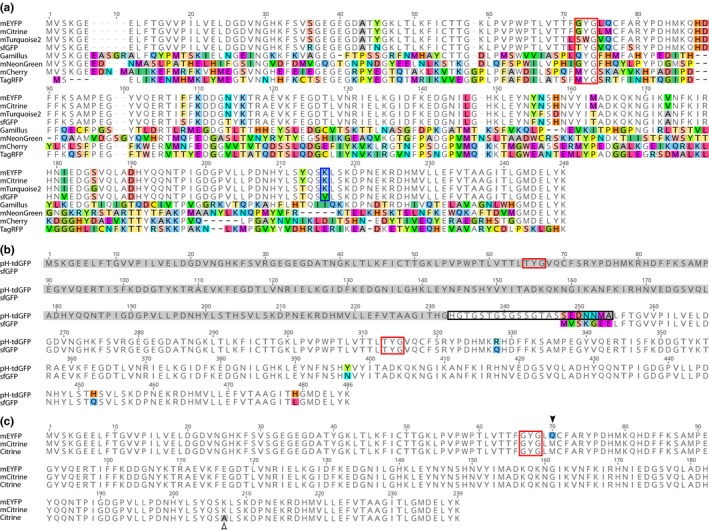
Multiple alignments of the 10 fluorescent proteins used in this study. (a) Is an alignment of all monomeric fluorescent tags tested, highlighting that proteins derived from *Aequorea victoria* (mEYFP, mCitrine, mTurquoise2 and sfGFP) are highly conserved while they are very different from the proteins derived from *Discosoma* sp (mCherry), *Entacmaea quadricolor* (TagRFP), *Branchiostoma lanceolatum* (mNeonGreen) and *Olindias formosa* (Gamillus). (b) Is an alignment of sfGFP and pH‐tdGFP. pH‐tdGFP comprises two copies of GFP (the N‐terminal copy is highlighted in gray), separated by a linker (black box). The N149Y and Q204H substitutions are responsible for increased pH stability in pH‐tdGFP while Q80R and L231H are neutral substitutions. (c) Is an alignment of the three yellow proteins (mEYFP, mCitrine and Citrine) showing the single amino acid substitutions responsible for increased pH stability (Q69M, black arrowhead) and monomerization (A206K, empty arrowhead). In all panels, non‐identity is indicated by coloring of amino acids and gaps in the alignment are depicted as dashes. All amino acid positions are calculated using the original *A. victoria* GFP sequence which lacked the V at position 2. Red boxes highlight the amino acids composing the chromophore, while the blue box highlights the amino acid responsible for the monomerization of the *A. victoria* FPs

A series of FPs with predicted superior pH stability and spanning the visible light spectrum were selected following two criteria. Each protein had to (a) be monomeric to avoid protein aggregation artifacts, and (b) have a low p*K*a (Table 1 and Figure 1a). A few pH‐sensitive FPs were also included, as negative controls.

In the blue/cyan range, mTurquoise2 was chosen for its very low p*K*a (p*K*a_mTurquoise2_ = 3.1), which was the lowest of all proteins tested in this study (Goedhart et al., 2012). Despite being a relatively new FP, it has been successfully expressed in several plant species such as *A. thaliana*,* N. benthamiana,* and *Marchantia polymorpha* (Boehm, Ueda, Nishimura, Shikanai, & Haseloff, 2016; Ghareeb, Laukamm, & Lipka, 2016; Rolland, Badger, & Price, 2016).

In the green range, pH‐tdGFP, sfGFP, Gamillus, and mNeonGreen were selected. The commonly used pH‐sensitive sfGFP (p*K*a_sfGFP_ = 5.9) was used as a negative control for its derivative pH‐tdGFP (p*K*a_pH‐tdGFP_ = 4.8), a protein with greater in vitro resistance to low pH (Pédelacq, Cabantous, Tran, Terwilliger, & Waldo, 2005; Roberts et al., 2016). Interestingly, the amino acid substitutions which reduce the pH sensitivity of pH‐tdGFP also revert the protein to an obligate dimer (Roberts et al., 2016). To prevent the formation of protein aggregates by dimerization of two GFPs in *trans*, Roberts et al. (2016) used two improved sfGFP molecules separated by a spacer, to allow the two GFPs to dimerize in *cis* (Figure 1b). To our knowledge, pH‐tdGFP has never been expressed in plants. Gamillus was recently isolated from *Olindias formosa* and has the lowest p*K*a of all published green proteins (p*K*a_Gamillus_ = 3.4), but like pH‐tdGFP has yet to be tested in plants (Shinoda et al., 2018). Finally, mNeonGreen was included because, despite its relatively high p*K*a (p*K*a_mNeonGreen_ = 5.7), it is one of the brightest fluorescent proteins available to date (Shaner et al., 2013).

Yellow proteins are notorious for their sensitivity to low pH and their relatively high p*K*a (Shaner et al., 2005; Young, Wightman, Blanvillain, Purcel, & Gallois, 2010). In this range, we selected mCitrine (p*K*a_mCitrine_ = 5.7) which has a p*K*a significantly lower than that of its parent protein mEYFP (p*K*a_mEYFP_ = 6.9), included as pH‐sensitive control (Figure 1c) (Griesbeck, Baird, Campbell, Zacharias, & Tsien, 2001; Kremers, Goedhart, van Munster, & Gadella, 2006; Miyawaki et al., 1999; Ormö et al., 1996; Rizzo, Springer, Granada, & Piston, 2004). To test the importance of the monomeric state of mCitrine, we introduced the K206A substitution to create a dimeric FP, Citrine (Figure 1c).

Finally, in the red range we selected TagRFP (p*K*a_TagRFP_ = 3.8) for its low p*K*a and relative brightness for a red protein, while the commonly used mCherry was selected for its stability and low p*K*a (p*K*a_mCherry_ < 4.5) (Merzlyak et al., 2007; Shaner et al., 2004).

### 3.2 Accurate protein targeting to the cytosol and the apoplast

**Figure 2:**
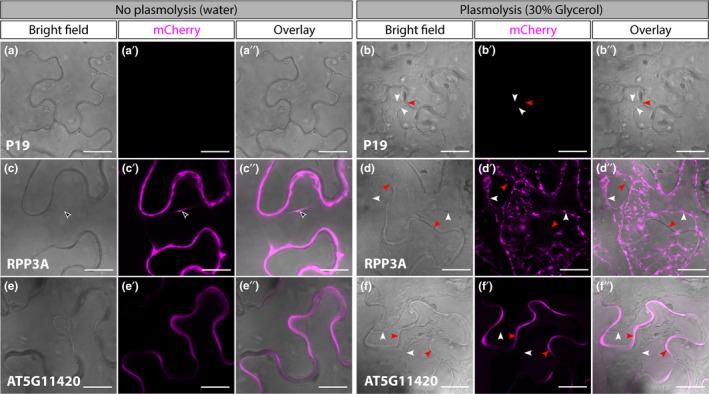
Confocal images showing the localization of mCherry targeted to the cytosol or the apoplast of *Nicotiana benthamiana* epidermal cells, 3 days post infiltration. Cells expressing P19 alone (a‐a″ & b‐b″), P19+ RPP3A‐mCherry (c‐c″ & d‐d″, mCherry localized to the cytosol), or P19 + AT5G11420‐mCherry (e‐e″ & f‐f″, mCherry localized to the apoplast) are shown in water (a‐a″, c‐c″, e‐e″) or plasmolyzed (b‐b″, d‐d″, f‐f″, leaf air space infiltrated with 30% (v/v) glycerol). Empty arrowheads highlight transvacuolar strands, white arrowheads point at the plasma membrane and red arrowhead show cell walls. All images are single planes. Scale bars represent 20 μm

To compare the performance of each FP in the cytosol and the apoplast, we agroinfiltrated leaves of *Nicotiana benthamiana* with constructs of interest and visualized transformed tissue 3 days post infiltration (3 dpi) using confocal microscopy. Two studies previously reported that RPP3A‐EGFP and AT5G11420‐TagRFP were localized to the cytosol and the apoplast, respectively (Albenne et al., 2014; Cutler et al., 2000). To confirm that RPP3A and AT5G11420 are able to target FPs to the cytosol and the apoplast, respectively, we analysed the subcellular distribution of RPP3A‐mCherry and AT5G11420‐mCherry with and without plasmolysis in a hypertonic solution (Figure 2). The retraction of the plasma membrane from the cell wall enabled separate visualization of the intracellular and extracellular compartments. In unplasmolyzed cells, RPP3A‐mCherry was targeted to the cytosol as demonstrated by the detection of mCherry fluorescence in transvacuolar strands (Figure 2c‐c″). After plasmolysis the mCherry fluorescence had moved away from the cell wall (Figure 2d‐d″), confirming that RPP3A directed mCherry to the cytosol. In contrast, AT5G11420‐mCherry fluorescence outlined the edges of cells, was absent from transvacuolar strands, and remained in the cell wall after plasmolysis, confirming that AT5G11420‐mCherry localized in the apoplast (Figure 2e‐e″ and 2f‐f″). These results confirmed the suitability of RPP3A and AT5G11420 to target FPs to the cytosol and the apoplast, respectively.

### 3.3 Comparing the relative fluorescence of FPs targeted to the cytosol or the apoplast

**Figure 3:**
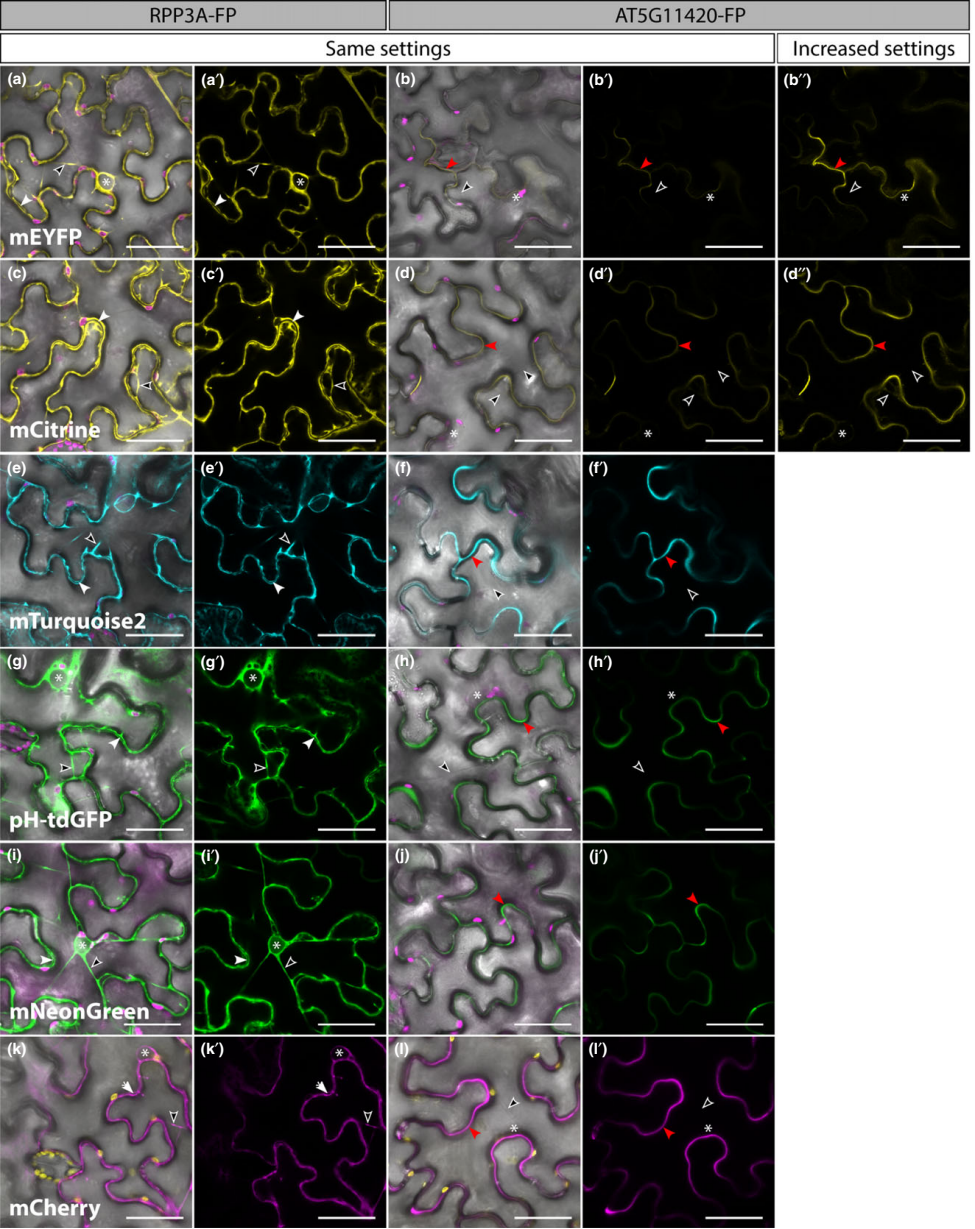
Confocal images of *Nicotiana benthamiana* epidermal cells (3 days post infiltration) showing the comparative intensity of RPP3A‐FP (first two columns) and AT5G11420‐FP (last three columns) for the fluorescent proteins which did not form aggregates in the cytosol. For each construct, the first four panels were imaged with the same imaging settings, while the 5th panel displays increased settings (e.g. increased laser intensity, detector gain or levels in Photoshop/LASX), when needed. RPP3A‐mEYFP (yellow in a‐a′), RPP3A‐mCitrine (yellow in c‐c′), RPP3A‐mTurquoise2 (cyan in e‐e′), RPP3A‐pH‐tdGFP (green in g‐g′), RPP3A‐mNeonGreen (green in i‐i′) and RPP3A‐mCherry (magenta in k‐k′) all localized in the cytosol and were visible in transvacuolar strands (empty arrowheads) and around nuclei (asterisks), while they were absent from the cell wall space (white arrowheads). RPP3A‐mNeonGreen was also detected inside nuclei (i‐i′). All AT5G11420‐FP fusions were detected in the apoplast (red arrowheads) but AT5G11420‐mEYFP (yellow in b‐b″) and AT5G11420‐mCitrine (yellow in d‐d″) were dim while AT5G11420‐mTurquoise2 (cyan in f‐f′), AT5G11420‐pH‐tdGFP (green in h‐h′), AT5G11420‐mNeonGreen (green in j‐j′), and AT5G11420‐mCherry (magenta in l‐l′) could easily be detected using the same settings as for their respective RPP3A‐FP fusion. Chloroplasts are shown in magenta (a–j) or yellow (k‐l). FP: Fluorescent Protein. All images are single planes. Scale bars represent 40 μm

**Figure 4:**
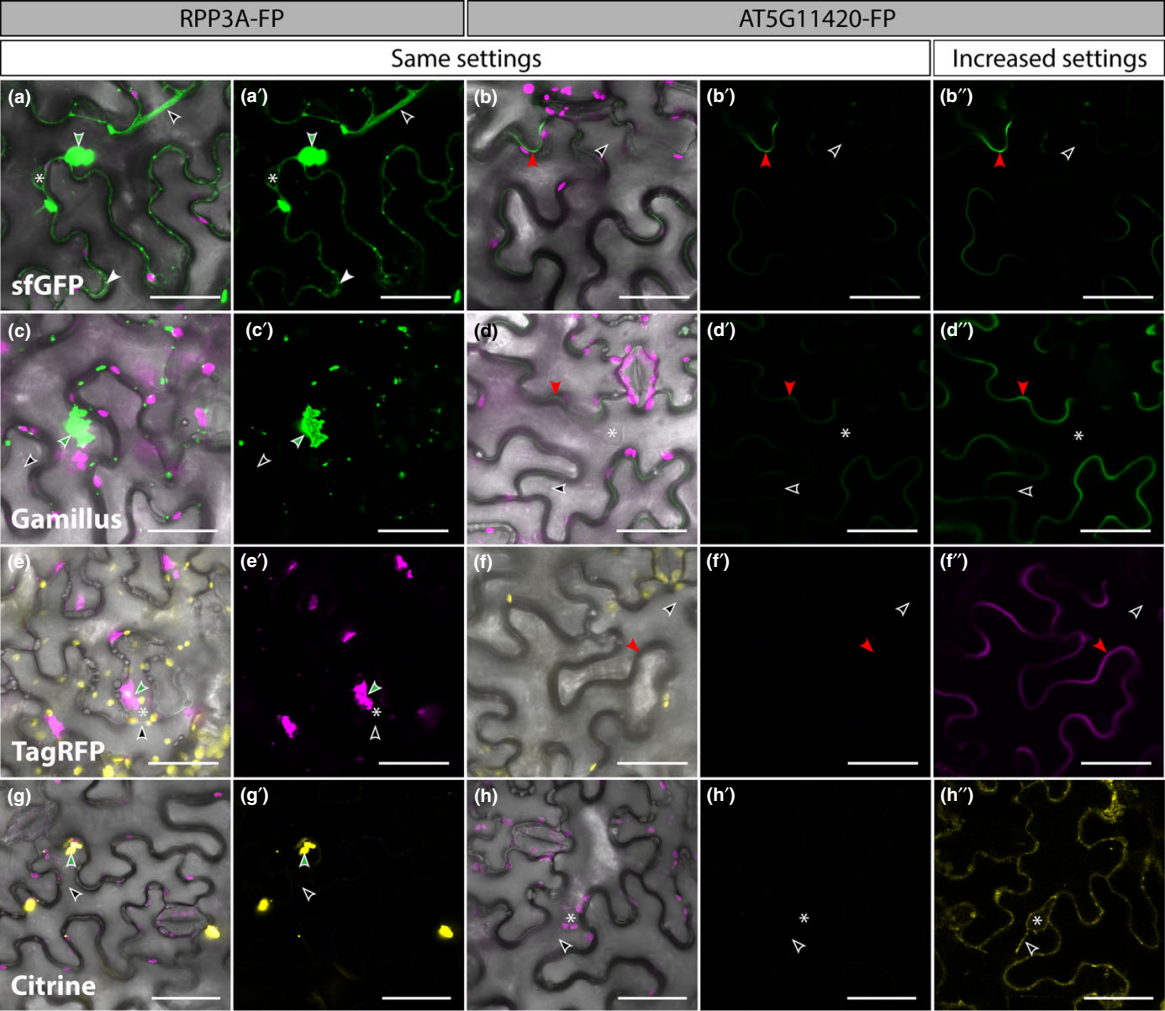
Confocal images of *Nicotiana benthamiana* epidermal cells (3 days post infiltration) showing the comparative intensity of RPP3A‐FP (first two columns) and AT5G11420‐FP (last three columns) for the fluorescent proteins which formed aggregates in the cytosol. For each construct, the first four panels were imaged with the same imaging settings, while the 5th panel displays increased settings (e.g. increased laser intensity, detector gain or levels in Photoshop/LASX). RPP3A‐sfGFP (green in a‐a′), RPP3A‐Gamillus (green in c‐c′), RPP3A‐TagRFP (magenta in e‐e′), and RPP3A‐Citrine (yellow in g‐g′) all formed bright aggregates in the cytosol (green arrowheads). RPP3A‐Gamillus (c‐c′), RPP3A‐TagRFP (e‐e′), and RPP3A‐Citrine (g‐g′) could not be found in transvacuolar strands (empty arrowheads) because the aggregates were too bright. Contrastingly, RPP3A‐sfGFP (a‐a′) could be detected in transvacuolar strands (empty arrowheads) and around the nucleus (asterisks), and was absent from the cell wall space (white arrowheads). The brightness of cytosolic aggregates meant that AT5G11420‐TagRFP (f‐f′), and AT5G11420‐Citrine (h‐h′) could not be detected when using the same settings as for their RPP3A‐FP respective fusion, while AT5G11420‐sfGFP (b‐b′) and AT5G11420‐Gamillus (d‐d') were very weak. However, increased settings revealed that AT5G11420‐sfGFP (b″), AT5G11420‐Gamillus (d″), and AT5G11420‐TagRFP (f″) localized in the apoplast (red arrowhead) and were absent from transvacuolar strands (empty arrowheads). Contrastingly, AT5G11420‐Citrine (h″) failed to reach the apoplast and was detected in transvacuolar strands (empty arrowheads) and around the nucleus (asterisks). Chloroplasts are shown in magenta in a, b, c, d, g and h and in yellow in e and f. FP: Fluorescent Protein. All images are single planes. Scale bars represent 40 μm

To determine whether or not a FP was able to fluoresce in the apoplast as well as in the cytosol, we transiently expressed each RPP3A‐FP and AT5G11420‐FP construct individually in *N. benthamiana* leaves, optimized the confocal settings on RPP3A‐FP and used the same settings to image the corresponding AT5G11420‐FP. In this experiment, FPs fell into two distinct categories depending on their behavior in the cytosol; some FPs distributed evenly in the cytosol (Figure 3) and some formed cytosolic aggregates (Figure 4).

#### 3.3.1 FPs which did not aggregate in the cytosol

RPP3A‐mEYFP (Figure 3a‐a′), RPP3A‐mCitrine (Figure 3c‐c′), RPP3A‐mTurquoise2 (Figure 3e‐e′), RPP3A‐pH‐tdGFP (Figure 3g‐g′), RPP3A‐mNeonGreen (Figure 3i‐i′), and RPP3A‐mCherry (Figure 3k‐k′) all localized evenly throughout the cytosol and were detected in transvacuolar strands, around nuclei, and were absent from the cell wall space. Additionally, RPP3A‐mNeonGreen was also detected inside nuclei, as is often the case with small cytosolic proteins. When fused to AT5G11420, mEYFP (Figure 3b‐b″), and mCitrine (Figure 3d‐d″) were hard to detect with the same settings as used for their cytosolic counterparts but were clearly observed in the apoplast with increased settings (e.g. increased laser intensity, detector gain, or levels in Photoshop/LASX). In cells expressing AT5G11420‐mEYFP and AT5G11420‐mCitrine, faint signal could also be detected inside the cells (Figure 3b″ and d″), which may reflect the “in transit” protein pool being secreted to the apoplast. In contrast, AT5G11420‐mTurquoise2 (Figure 3f‐f′), AT5G11420‐pH‐tdGFP (Figure 3h‐h′), AT5G11420‐mNeonGreen (Figure 3j‐j′), and AT5G11420‐mCherry (Figure 3l‐l′) were easily detected in the apoplast using the same settings as for their cytosolic counterparts, suggesting that these four tags may be good tools to track proteins in the apoplast of epidermal cells.

#### 3.3.2 FPs which formed aggregates in the cytosol

RPP3A‐Citrine was used as a positive control for aggregation as Citrine forms dimers (Figure 4g‐g′). Indeed, RPP3A‐Citrine formed cytosolic aggregates which were so bright that no signal could be detected in transvacuolar strands (Figure 4g‐g′). Despite being monomeric FPs in vitro, we found that sfGFP (Figure 4a‐a′), Gamillus (Figure 4c‐c′), and TagRFP (Figure 4e‐e′) formed bright aggregates in the cytosol when fused to RPP3A, indicating that they may form dimers in this context. Unlike Gamillus and TagRFP, sfGFP also localized evenly throughout the rest of the cytosol (Figure 4a‐a′). Interestingly, AT5G11420‐sfGFP (Figure 4b‐b″), AT5G11420‐Gamillus (Figure 4d‐d″), and AT5G11420‐TagRFP (Figure 4f‐f″) were correctly targeted to the apoplast where they did not seem to aggregate, although the intense brightness of their cytosolic aggregates meant that increased settings had to be used to detect them. In contrast, when the dimeric FP Citrine was fused to AT5G11420 it was detected in transvacuolar strands and around nuclei, suggesting a cytosolic localization (Figure 4h‐h″).

Taken together, our targeting experiments revealed that apart from Citrine all FPs could be targeted to the apoplast. Additionally, mTurquoise2, pH‐tdGFP, mNeonGreen, and mCherry did not aggregate in the cytosol and were detectable in both compartments with the same settings, suggesting that they are promising tags to track extracellular proteins.

### 3.4 Comparative sensitivity of cell wall‐targeted FPs across a pH gradient

**Figure 5:**
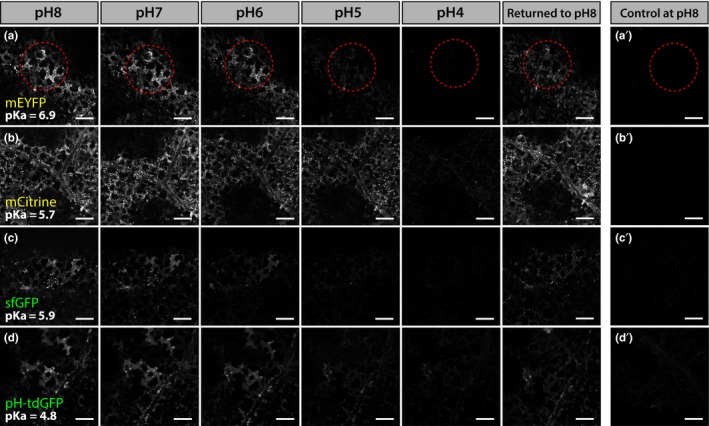
Confocal images of *Nicotiana benthamiana* spongy mesophyll cells (3 days post infiltration) showing the in vivo fluorescence of apoplastic mEYFP (a), mCitrine (b), sfGFP (c), and pH‐tdGFP (d) subjected to a pH 8–4 gradient. Fluorescent proteins were targeted to the apoplast by fusion with AT5G11420. For each construct, the same area of exposed spongy mesophyll was imaged in a pH 8 buffer, which was then replaced with buffers of gradually more acidic pH (in increments of 1 unit at a time, down to pH 4), before being returned to pH 8 to determine whether the fluorescence could be restored. For a given FP, all images were obtained by using the same imaging settings. a′, b′, c′ and d′ are images of tissue expressing P19 alone placed in a pH 8 buffer and imaged with the same imaging settings as a, b, c and d, respectively. The red disks in a and a′ highlight the tissue area which was used to quantify fluorescence variations across the pH gradient in the same group of cells (see Figure 7) and are provided as an example. All images are single planes. Scale bars represent 200 μm

**Figure 6:**
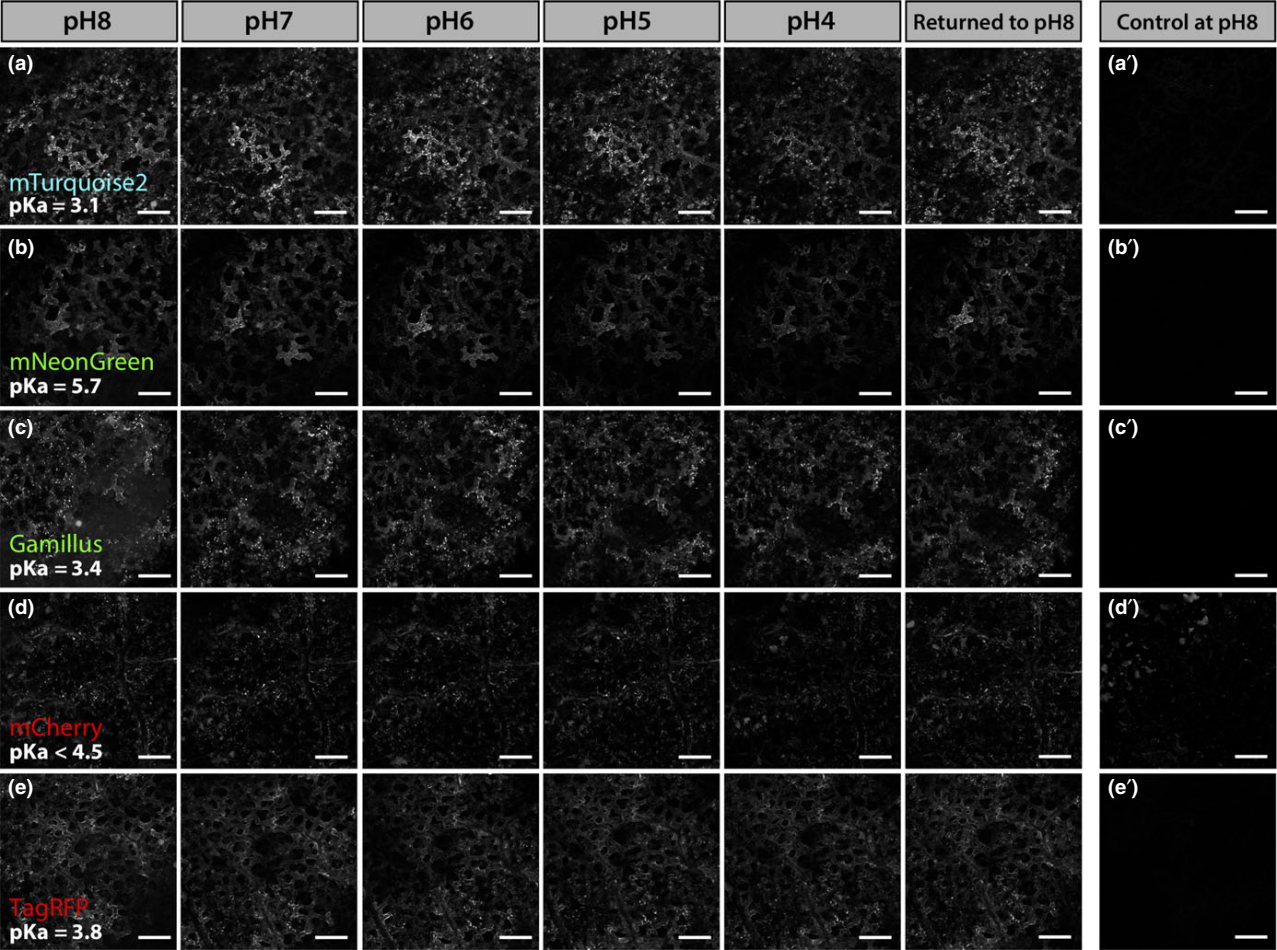
Confocal images of *Nicotiana benthamiana* spongy mesophyll cells (3 days post infiltration) showing the in vivo fluorescence of apoplastic mTurquoise2 (a), mNeonGreen (b), Gamillus (c), mCherry (d), and TagRFP (e) subjected to a pH 8–4 gradient. Fluorescent proteins were targeted to the apoplast by fusion with AT5G11420. For each construct, the same area of exposed spongy mesophyll was imaged in a pH 8 buffer, which was then replaced with buffers of gradually more acidic pH (in increments of 1 unit at a time, down to pH 4), before being returned to pH 8 to determine whether the fluorescence could be restored. For a given FP, all images were obtained by using the same imaging settings. a′, b′, c′, d′ and e′ are images of tissue expressing P19 alone placed in a pH 8 buffer and imaged with the same imaging settings as a, b, c, d and e, respectively. All images are single planes. Scale bars represent 200 μm

**Figure 7:**
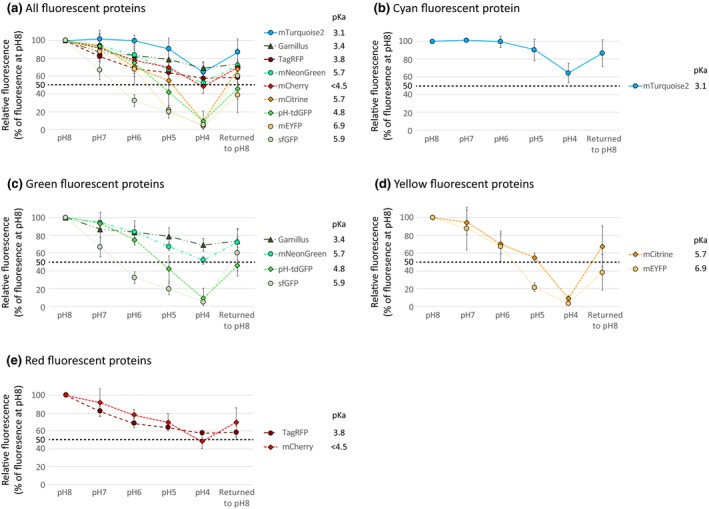
Quantification of the relative in vivo fluorescence of apoplastic mTurquoise2, sfGFP, pH‐tdGFP, Gamillus, mNeonGreen, mEYFP, mCitrine, TagRFP and mCherry across a pH 8–4 gradient. (a) Is an aggregation of the data for all fluorescent proteins tested and shows that at pH 4 there are two distinct groups of proteins: those which show less than 10% of their pH 8 fluorescence value (sfGFP, pH‐tdGFP, mEYFP, mCitrine, see Figure 5) and those which retain about or >50% of their pH 8 fluorescence (mTurquoise2, Gamillus, TagRFP, mNeonGreen, and mCherry, see Figure 6). The data is also presented per protein color (Cyan in b, Green in c, Yellow in d and Red in e). Each data point represents the mean fluorescence of 3 replicates and error bars represent standard deviation

Apoplastic pH is highly dynamic and the baseline extracellular pH of the epidermal cells tested here is unknown. Regardless of its exact value, it is unlikely to reflect the pH range that apoplast‐targeted FPs may be exposed to in other tissues/species. To test the in vivo behavior of apoplast‐targeted FPs across a range of pH values, we followed the fluorescence of AT5G11420‐FP‐expressing spongy mesophyll cells immersed in buffered solutions of progressively decreasing pH (pH 8 to pH 4, in increments of 1 pH unit), using the same instrument settings (Figures 5 and 6). The signal emitted by each FP was quantified at each pH value and normalized to the signal recorded in the pH 8 solution (Figure 7 and Table 2). In these experiments, FPs fell into two distinct categories depending on the fluorescence intensity left in the pH 4 buffer.

**Table 2:**
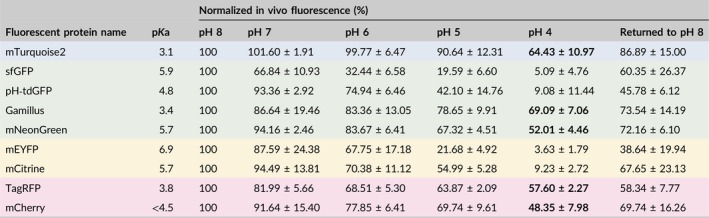
In vivo FP fluorescence in the same groups of cells across a pH series. This table summarizes the data presented in Figure 7, where each data point represents the mean fluorescence of 3 replicates ± standard deviation. Values in bold in the pH 4 column highlight the FPs which retained ~>50% of their pH 8 fluorescence (mTurquoise2, Gamillus, mNeonGreen, TagRFP and mCherry). The color of a row indicates the color range of the light emitted by the fluorescent protein

#### 3.4.1 FPs which did not perform well at low pH

This category of proteins contains the yellow pair mEYFP/mCitrine and the green pair sfGFP/pH‐tdGFP. Despite the improved FP of each pair performing better than its ancestor, all four proteins showed a gradual decrease in fluorescence across the pH gradient (Figures 5, 7 and Table 2). In the yellow pair, mEYFP and mCitrine performed similarly at pH 7 and pH 6, but the improved mCitrine was brighter at pH 5 (~55%) than its ancestor mEYFP (~21%) (Figure 5a‐b, 7d and Table 2). In the green pair, the improved pH‐tdGFP was brighter than its ancestor sfGFP from pH 7 (pH‐tdGFP: ~93%; sfGFP: ~66%) to pH 5 (pH‐tdGFP: ~42%; sfGFP: ~19%) (Figures 5c‐d, 7c and Table 2). However, in the pH 4 buffer all four proteins retained less than 10% of their original pH 8 fluorescence (Figures 5, 7c‐d and Table 2). To ensure that loss of signal at low pH was not due to FPs being washed out of the apoplast, or denatured, each peeled disk was placed back into a pH 8 solution after the final pH 4 treatment. In all cases fluorescence increased (~38% for mEYFP, ~45% for pH‐tdGFP, ~60% for sfGFP, and ~67% for mCitrine), indicating that fluorescence loss in the pH 4 buffer was transient and could be partially restored by increasing the pH (Figures 5, 7 and Table 2). These results suggest that mEYFP, mCitrine, sfGFP, and pH‐tdGFP are unsuited to low pH conditions in vivo.

#### 3.4.2 FPs which performed well at low pH

This category of proteins contains mTurquoise2, Gamillus, mNeonGreen, TagRFP, and mCherry. For these proteins, the decrease in FP signal over the pH gradient was more limited and their fluorescence in the pH 4 buffer was about or greater than 50% of their pH 8 fluorescence, ranging from ~48% for mCherry to ~69% for Gamillus (Figures 6, 7 and Table 2). FP fluorescence could be partially restored by placement in a pH 8 buffer at the end of each pH series (Figures 6, 7 and Table 2). These results show that mTurquoise2, Gamillus, mNeonGreen, TagRFP, and mCherry are able to withstand low pH conditions, in vivo.

## 4 DISCUSSION

### 4.1 Fluorescence inhibition and pH dynamics

All of the fluorescent proteins tested here were affected in vivo when the pH dropped, although to different degrees. Fluorescence inhibition at low pH was partially reversible and could be restored by returning the tissue to a pH 8 buffer (Figures 5, 6, 7 and Table 2). Fluorescence could be restored to high levels (e.g. ~86% for mTurquoise2) or restored from very low levels (e.g. ~5% to ~60% for sfGFP, or ~9% to ~67% for mCitrine). However, it was never restored back to 100% which is in line with previous experiments using GFP‐S65T and EYFP, presumably because protein conformation changes induced at pH < 5 require longer recovery times (Kneen, Farinas, Li, & Verkman, 1998; Young et al., 2010). Notably, the fluorescence of two proteins could not be restored even to 50% of the initial values: mEYFP (~38%) and pH‐tdGFP (~45%). Why their fluorescence recovery was so poor remains unclear. We note that mEYFP has the highest p*K*a of all proteins tested in this study (6.9) and that pH‐tdGFP is a tandem dimer. It is possible that these characteristics contribute to mEYFP and pH‐tdGFP being more sensitive than other FPs to long‐term conformational changes induced by low pH.

### 4.2 Protein aggregation and nomenclature

The weak dimer Citrine was used as a positive control to test the effect of dimerization on protein targeting in the cytosol. As expected, very bright aggregates were detected in the cytosol of cells expressing RPP3A‐Citrine (Figure 4g‐g′). When fused to AT5G11420, Citrine could not be detected in the apoplast and instead was retained inside the cells (Figure 4h‐h″). It is unclear whether Citrine dimerization inhibited proper apoplast targeting or whether the part of the protein pool that was exported to the apoplast was unable to fluoresce. In the cytosol of epidermal cells, sfGFP, Gamillus, and TagRFP formed bright aggregates, suggesting that they also dimerize, *in planta* (Figure 4a‐a′, c‐c′, e‐e′). This is surprising because although Gamillus had never been tested in plants before, all three FPs are monomeric in vitro (Merzlyak et al., 2007; Pédelacq et al., 2005; Shinoda et al., 2018). In cells expressing RPP3A‐Gamillus or RPP3A‐TagRFP, the aggregates were so bright that no diffuse signal was detected in the cytosol, but RPP3A‐sfGFP was also detected diffusely throughout the cytoplasm, suggesting that sfGFP may be a weaker dimer than Gamillus and TagRFP. The fact that FPs were fused to RPP3A rather than being free in the cytosol may have enhanced the aggregation of these three proteins. However, fusion to RPP3A did not affect any of the other FPs, which indicates that Gamillus, TagRFP and sfGFP are more prone to forming aggregates in these conditions than any other FP tested in this study. Interestingly, sfGFP, Gamillus, and TagRFP efficiently localized in the apoplast when fused to AT5G11420, suggesting that even if they do form aggregates in some situations, they may be suitable for labeling proteins targeted to the apoplast (Figure 4b‐b″, d‐d″, f‐f″).

In FPs related to EGFP, monomerization is encoded by a single amino acid at position 206. For example, the monomer mCitrine can be obtained from the weak dimer Citrine through a single substitution (A206K, Figure 1c). The same amino acid substitution is present in other monomeric FPs derived from *Aequorea victoria* such as mEYFP and mTurquoise2 (Figure 1a). Interestingly, the substitution making sfGFP a monomer is A206V (Figure 1a). While sfGFP crystallizes as a monomer, it is possible that A206V is not as efficient as A206K at preventing dimerization in plants, under certain conditions. It is difficult to assess which amino acids are involved in monomerization in TagRFP and Gamillus because these proteins were isolated from different organisms and their sequences are too dissimilar for comparison with FPs isolated from *A. victoria* (Figure 1a). Nevertheless, our results with Citrine/mCitrine and sfGFP highlight the consequences of single amino acid substitutions in FPs and hence the need for accuracy in the nomenclature used to refer to specific FPs.

### 4.3 In vitro properties only partially predict in vivo performance

Most of our findings could be predicted from the in vitro properties of the tags tested. For example, our analysis of protein pairs (mEYFP/mCitrine and sfGFP/pH‐tdGFP) showed that FPs engineered to be more pH stable (pH‐tdGFP and mCitrine) did indeed retain more fluorescence at low pH than their ancestors (sfGFP and mEYFP). We also found that the four proteins with the lowest p*K*a, mTurquoise2, Gamillus, TagRFP, mCherry, resisted low pH well in vivo. However, in vitro protein properties did not always correlate with in vivo protein performance. For example, one of the most pH‐insensitive proteins in this study was mNeonGreen, which has the same p*K*a as mCitrine (5.7) but performed much better at low pH than the yellow protein; mNeonGreen fluorescence was only partially reduced at pH 4 (~52% of its pH 8 fluorescence) whereas mCitrine fluorescence was severely reduced at the same pH (less than 10% of its pH 8 fluorescence) (Figure 7 and Table 2). Conversely, even though pH‐tdGFP has a lower p*K*a than mNeonGreen (p*K*a_pH‐tdGFP_ = 4.8), in a pH 4 buffer pH‐tdGFP retained less than 10% of its pH 8 fluorescence (Figure 7 and Table 2). Our study provides some clarity as to which FPs can withstand low pH in vivo, but the FP must be tailored to an experiment's needs.

### 4.4 Choosing the right FP: some considerations

Choosing the best FP for an experiment depends on many parameters, which include the particular constraints of the subcellular compartment being targeted (e.g. its pH), the fluorescence overlap with a stain or another FP, as well as the specifications of the imaging equipment available (e.g. microscope type, lasers, filters, detectors). Another important consideration is the relative brightness of a protein over autofluorescence, which can vary depending on the tissue/species. Many plant tissues/structures such as cell walls and cuticles are strongly autofluorescent in the blue, green and red range. Mitigating autofluorescence can be achieved in different ways, depending on the situation. For example, a standard confocal microscope cannot produce the excitation maximum (434 nm) of the blue/cyan protein mTurquoise2, and we found that excitation at 458 nm, rather than 405 nm, increased the FP/background ratio. This had important implications because mTurquoise2 was the second dimmest protein of our set. Choosing the most suited laser (458 nm) meant that this FP could be easily detected above background in all experiments, and was one of the best performing FPs of our study overall (Tables 1 and  3). In the epidermis, another approach was to avoid imaging the waxy surface of the cells, focusing instead on slightly more internal planes. The pH series experiments in spongy mesophyll cells, however, were considerably more difficult to image due to the combination of air/cell/buffer interfaces around these cells and high tissue autofluorescence, in particular in the green and red parts of the spectrum. In the green range, even when proteins were very bright (i.e. mNeonGreen and Gamillus) autofluorescence was problematic, while the red proteins TagRFP and mCherry are not very bright to start with (Table 1). One approach we used to deal with background fluorescence was to time‐gate detectors, a method which can eliminate reflection for highly refractive interfaces and autofluorescence without affecting the signal coming from a FP (Kodama, 2016). However, not all confocal detectors can be time‐gated, so autofluorescence cannot always be addressed this way. In any case, autofluorescence is a critical factor to take into consideration when choosing the best FP for a given experiment.

## 5 SUMMARY AND CONCLUSION

The broad pH range (3.5–8.3) experienced by proteins in the apoplast, combined with the rapid pH changes induced by hormones, cell growth, and biotic and abiotic stresses, calls for the use of robust fluorescent tags able to withstand these dynamics (Arsuffi & Braybrook, 2018; Barbez et al., 2017; Geilfus, 2017; Yu et al., 2000). There are well over 100 FPs available to date, but there is no silver bullet to determine the best FP for any given experiment without trial and error. In this study, we investigated the behavior of 10 FPs, including pH‐tdGFP and Gamillus which had never been expressed in plants before, and our results suggest that FPs can be split into three categories (Table 3). sfGFP, pH‐tdGFP, mEYFP, Citrine/mCitrine were deemed inappropriate for the apoplast because they were not able to withstand low pH conditions in vivo (or could not be properly targeted to the apoplast in the case of Citrine). Gamillus and TagRFP were deemed fit for purpose under certain conditions because they were able to withstand low pH but formed aggregates in the cytosol, which suggests that they could be problematic in some situations. Finally, mTurquoise2, mNeonGreen, and mCherry performed well overall and we suggest that they are suitable to track proteins in the cell wall under dynamic pH conditions. Our results demonstrate that the in vivo functionality of a given FP is underpinned by the combination of its in vitro characteristics, the properties of the tissue in which it is to be expressed and the constraints of the imaging equipment available. Consideration of the combination of these factors will certainly assist in streamlining and optimizing the design of FP‐based experiments in plant tissue.

**Table 3:**
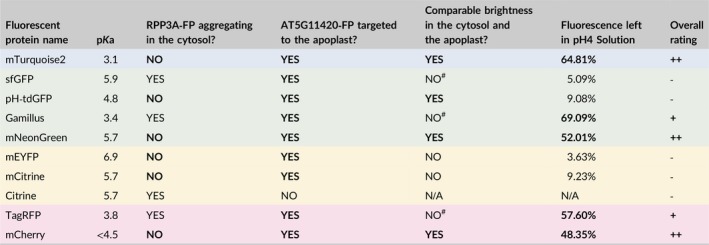
Summary of in vivo properties of the fluorescent proteins tested in *Nicotiana benthamiana* leaves. ^#^Comparison of detection levels was biased by the fact that sfGFP, Gamillus and TagRFP formed bright aggregates in the cytosol when fused to RPP3A. Desirable results in each category are highlighted in bold. Proteins which were targeted to the apoplast when fused to AT5G11420 and which retained about or >50% of their maximum in vivo fluorescence (as measured in a pH 8 solution) when placed in a pH 4 solution were given an overall rating of “++” (when RPP3A‐FP did not aggregate in the cytosol) or a “+” (when RPP3A‐FP aggregated in the cytosol). All other proteins were given an overall rating of “‐”. The color of a row indicates the color range of the light emitted by the fluorescent protein

## AUTHOR CONTRIBUTIONS

V.R. conceived the research; A.S. and V.R. performed the research; A.S. and V.R. analysed the data; V.R. drafted the manuscript; A.S. and V.R. contributed to the final manuscript.
