# Supplementary material for: I see the light! Fluorescent proteins suitable for cell wall/apoplast targeting in Nicotiana benthamiana leaves
Source: Plant Direct. 2019 Jan 17;3(1):e00112. doi: 10.1002/pld3.112 (PMC6508812; doi:10.1002/pld3.112)
Supplement: Supplementary file 1 [file PLD3-3-e00112-s001.pdf]

RE: I see the light! Fluorescent proteins suitable for cell wall/apoplast targeting

Dear Dr. Rolland:

Thank you for submitting to Plant Direct. All required reviews have been returned and we have now finished our evaluation of your manuscript. In light of the reviewers' comments, further revisions are needed before the paper can be accepted for publication in Plant Direct.

Please view the reviewers' comments below and use their suggestions as a guide while you work on your revision. A major issue raised by both reviewers is that fluorescence intensity information should be provided and the claimed fluorescence intensity changes should be compared to a standard. We strongly suggest that you adopt Reviewer 1's suggestion of co-infiltrating with mCherry. In response to reviewer 1's second comment, we would like to request that you clearly indicate in the title and in the abstract that the experiments were performed using tobacco leaves, i.e, try to limit your conclusion to things you have tested. As for reviewer 1's first comment of testing more proteins, we welcome the experiments but they are not required for the manuscript to be accepted.

When uploading the revised version of this article, please be sure to include the following:

-A word document that contains your response to the reviewers. You should respond to each reviewer comment and note the changes made to the manuscript. If you do not agree with a reviewer's comment and choose not to make a suggested revision, please explain why. Please try to provide as complete an answer as possible to each reviewer's criticisms.
-A tracked changes document with each change highlighted
- A clean version of the latest version of the manuscript

To upload your revision, please click the link below.
https://plantdirect.msubmit.net/cgi-bin/main.plex?el=A5Lr2EA7A3Hdx5I1A9ftdtLKBIF3h696W3Y83NB97PQZ

In order to provide as timely a service as possible, we ask that your revision is resubmitted within three months after receipt of this request. If an extension is needed, please send a request, along with a brief explanation, to the editorial office at plantdirect@wiley.com .

Please note that, in addition to publishing reviewer comments, the author's responses to review comments will also be published alongside the final version of the paper. If you would not like the author's responses to be published, please contact the editorial office at plantdirect@wiley.com .

Thank you very much for giving us an opportunity to review your work. I look forward to receiving the next version.

Sincerely,

Alexander Schulz

Hsou-min Li

Editor, Plant Direct

<u>General comments to the Editors and Reviewers</u>

We would like to thank Reviewer 1 and Reviewer 2 for their constructive reviews and their thoughtful suggestions to improve our manuscript. We have responded to each comment made by the reviewers (see detailed responses below). In particular, we have followed Reviewer 1's suggestion to test 2 fluorescent proteins – we have gone even further and tested 3 new fluorescent proteins (FPs). We have also addressed the request made by both reviewers to quantify the fluorescence of our pH series. While this does not change our conclusions, we believe that it greatly strengthens our conclusions and the impact of our study. We hope that you will agree that it is now suitable for publication in Plant Direct.

These significant additional experiments have resulted in the following changes:
- We have added 3 full figures and 2 new tables (our manuscript now contains 7 figures and 3 tables). Former figures 4 and 5 have both been split into two (now called Figure 3, Figure 4, Figure 5 and Figure 6) to include the results of the 3 new FPs. Figure 7 and Table 2 are completely new and show the quantification of the fluorescence for all FPs across our pH series. Finally, have included a summary of all our results in a new table (Table 3).
- Most sections of the manuscript have been updated to include these new experiments and address the Reviewer's comments, and the Results and Discussion sections have been very largely re-written for the same reason.
- We have also changed the title of our manuscript, as suggested by the Editors.

At the end of the manuscript, we have included a "Summary and conclusion" paragraph which briefly summarizes the main findings of our manuscript. Briefly, we found that all FPs tested in our study can be grouped into three categories:
- sfGFP, pH-tdGFP, mEYFP, Citrine/mCitrine were deemed inappropriate for the apoplast because they were not able to withstand low pH conditions in vivo (or could not be properly targeted to the apoplast in the case of Citrine).
- Gamillus and TagRFP were deemed fit for purpose under certain conditions because they were able to withstand low pH but formed aggregates in the cytosol, which suggests that they could be problematic in some situations.
- Finally, mTurquoise2, mNeonGreen and mCherry performed well overall and we suggest that they are suitable to track proteins in the cell wall under dynamic pH conditions.

Below is a detailed response to each comment made by the Reviewers.

<u>Reviewer #1:</u>

Due to differences in the PKa values of fluorescent proteins, their application in tissues with changing or low pH is not always straightforward. This work describes an analysis of the functionality of a subset of fluorescent proteins in the low pH apoplast of Tobacco (N.bentamiana) leaves. This is, in principle, a very useful analysis that can help to determine which fluorophore to choose before committing to a time-consuming transformation and analysis. However, I think the current stage of this work is preliminary and would need additional experiments. In addition, there are a number of smaller issues which needs to be addressed before a possible publication.

Major issues

1) In l. 325-335 p. 11 the authors discuss the applicability of the mNeonGreen fluorophore. This protein represents a newer generation of fluorophores together with the synthetic red monomer mScarlet designed for acidic environments. These proteins are publically available and should be included in this analysis.

This is a great suggestion. To address this comment, we have tested three new FPs, rather than two! Our manuscript now includes tests for 10 FPs.
We chose to test the two green proteins which we discussed in the previous version of our manuscript, mNeonGreen and Gamillus, as well as the red protein TagRFP.
- Gamillus has a very low pKa (3.4) but had never been tested in plants as it was published a few months ago. We found that it worked very well in the apoplast upon acidification, and we suggest that it could be used in the apoplast but may not be suitable for all applications given that it formed aggregates in the cytosol.

- mNeonGreen has a higher pKa (5.7) but is to our knowledge the brightest monomeric green protein available to date. We found that it behaved well in all our experiments and we consider it one of the best proteins tested in our study.
- finally, we tested TagRFP rather than mScarlet because of its significantly lower pKa (3.8 for TagRFP versus 5.3 for mScarlet). We hypothesized that TagRFP would be more pH resistant than mScarlet. In fact, TagRFP behaved similarly to Gamillus (although it was much dimmer) and we suggest that it could be used in the apoplast but may not be suitable for all applications given that it formed aggregates in the cytosol.

We would like to point out that while these proteins are publicly available, we decided to clone them ourselves to ensure that all constructs tested in our study included the same linkers, etc, to allow for direct comparisons between all FPs.

As noted in our general comments above, the addition of these three proteins has resulted in a modification of the text and figures (3 new figures and 2 new tables). We are grateful to reviewer 1 for their suggestion as we believe that is has contributed to greatly improving our manuscript.

2) The authors mention the fluctuating pH environment of the Arabidopsis root (l 75-78 p. 3), yet chooses to work with transient expression in tobacco as it is an easier system for transformation. However, the Arabidopsis meristematic tissue to represent a useful model to study fluctuations in pH as different zones might have very distinct pH values . - It would be extremely useful for a much broader audience with an analysis of the behavior of fluorescent reporters this tissue in addition to the transient tobacco system, especially since the mentioned issues with autofluoresence is much greater in the cell walls of the arabidopsis meristem than in tobacco epidermis, especially in the blue range where mTurquise2 is currently the best option.

We agree that it would be interesting to test how these proteins behave in different species/tissues. However, to our knowledge our study is the first to compare the behaviour of 10 different fluorescent proteins across a pH gradient, *in vivo*. We believe that investigating the behaviour of these proteins in roots should be the focus of a separate study. We have, however, modified the title of the manuscript and the abstract to reflect the fact that we have tested these proteins in *N. benthamiana* leaves, as requested by the Editors.

We would also like to point out that pH is highly dynamic in a whole range of cell types/species, not just roots of Arabidopsis. To clarify this, we have modified the parts of the introduction which deals with measurements of pH dynamics. We have also added a new section to the discussion, in which we discuss tissue autofluorescence and various ways we dealt with it - including when imaging mTurquoise2. The maximum excitation for mTurquoise2 is at 434nm, which is not possible with a standard confocal microscope. We found that using a 458nm laser, rather than a 405nm laser, increased the FP/background ratio. This had important implications because mTurquoise2 is the second dimmest protein of our set but it could easily be detected above background in all experiments and ended-up being one of the best performing FPs of our study, overall. This may also be useful in roots?

3) The figures depicting the comparative intensity of the diverse fluorophores (figure 4 and 5?) needs a more intensity-sensitive LUT. For example RGB or rainbow LUTs that allow a direct assessment of the relative intensity.

We decided to stick to grayscale because other LUTs can be problematic for colour-blind people. Colour-blindness affects a significant portion of readers and is not limited to red/green colour-blindness - it can affect various parts of the visible spectrum. However, we have now included a new figure (Figure 7) and a new table (Table 2) which summarize the fluorescence quantification we have performed. We believe that this figure and table are a much more robust way to assess fluorescence inhibition by pH.

Also, I think the manuscript would benefit from showing quantification of the relative intensities in the different experiments.

This is a fantastic suggestion, which was also made by reviewer 2.

Quantifying the fluorescence in the experiments where we expressed the FPs in the cytosol and the apoplast (now Figure 3 and Figure 4) would not be meaningful because they are done in different cells and even different

compartments. These experiments are meant as a rough indication as to whether or not the same FP can be detected in these 2 compartments using the same confocal settings.

However, quantifying the fluorescence in our pH series is a very valuable experiment because it can be done in the same group of cells across the pH series and allow for relative comparisons to be made. Three pH series were quantified for each one of the 9 FPs tested (a total of 27 pH series - Citrine was not tested because we could not detect it in the apoplast). This quantification is presented in a new figure (Figure 7) and a new table (Table 2). We have also added a new paragraph to the Mat and Met section to explain how we quantified the fluorescence. In short, to account for slight tissue rotation induced by the repeated replacement of buffered solutions, the cumulative fluorescence value (called RawIntDen in Image J) of a disc of 512 µm in diameter was measured in each image. The chlorophyll channel (not shown) was used to ensure that discs were located over the same group of cells across a pH series. Background fluorescence (as measured in a disc of 512 µm in diameter expressing P19, placed in 50mM HEPES pH 8, and imaged with identical settings) was deducted from each measurement in a given pH series. Fluorescence intensities were then normalised to the fluorescence measured at pH 8 (arbitrarily set at 100%).

The results of this quantification show that fluorescent proteins fall into two categories:
- mTurquoise2, Gamillus, mNeonGreen, TagRFP, mCherry retained ~>50% of their pH 8 fluorescence when placed in a pH 4 buffer. This group can further be split in two, depending on whether or not they aggregated in the cytoplasm (see general comment above).
- sfGFP, pH-tdGFP, mEYFP, mCitrine retained <10% of their pH 8 fluorescence when placed in a pH 4 buffer. These proteins are considered unsuited to the apoplast.

We are extremely grateful to both Reviewers for this suggestion as we believe it has made our manuscript much stronger!

4) As the level of expression is not directly addressed within each analysis, a quantification of the amount of expression is needed by either WB or qPCR. Alternatively, I would consider it fine if the authors could simply co-infiltrate the tested fluorophores with mCherry to address the relative disappearance of signal compared to a more pH stable fluorescence.

Quantifying RNA would not provide information about protein expression, and the results of a WB performed on one or several whole disc would be hard to relate to those obtained from a smaller group of cells being imaged. Additionally, protein expression and protein detection are two different things – detection also depends on protein brightness and tissue autofluorescence, amongst others. A western blot would not take this into account. Finally, we did not have any leaf material left that could be linked to our experiments (and these pH series are quite tedious to perform in the first place).

The idea of co-expressing a pH stable and a pH resistant protein in the same leaf is an interesting one. However, it is not a trivial to perform, especially because of a technical challenge: all relevant protein pairs have overlapping spectra which suggests a significant chance of generating FRET – which would render any fluorescence quantification difficult to interpret as signal could be transferred from one FP to another.

However, we have now quantified the fluorescence of each FP across a pH gradient (Figure 7 and Table 2). This provides solid measurements of the fluorescence of a given FP in the same group of cells – irrelevant of their expression level. We have normalised the fluorescence to its value in a pH 8 buffer, which also allows for FPs to be directly compared – we believe that this is the most solid way of comparing FPs.

5) Another way to improve the manuscript would be to include a known pH sensor to confirm the actual apoplastic pH under pH treatments.

This is an interesting point, which is also highly debated one in the field! All pH sensors we are aware of need to be calibrated against a pH series (which is the same as what we have done here) and it is hard to prove that a buffer has completely obliterated the buffering capacity of a compartment. We are not claiming that in a pH 4 buffer, the pH of the cell wall was exactly 4 (it could be 4.2, for example, rather than 4). Instead, our study is comparative: all proteins were expressed in the same cell types, at the same stage, we used the same buffers in all experiments, and the fluorescence measured in the same cells was normalised to its value at pH 8. This

alleviates the need for such an experiment (also see comment in response to point 4, above). However, we have revised the text to make sure that we do not claim that at pH 4, the cell wall pH was exactly 4.

minor issues

• L. 117-120 p.4 : A reference is missing. what is the evidence for 4xGly addition to the C-terminal prevent mislocalization. Also, does this affect the spectra of the fluorophore?

This is a good point. We have now added a reference to support this claim (Nishizawa et al., 2003). We are not aware that it affects FP spectra and all FPs were cloned the exact same way.

• L. 140 P.5: what is P19? Why is it added? More information is needed.

Good point – we have added a sentence explaining what P19 is and why we used it, together with a reference: A vector encoding the tomato bushy stunt virus P19 protein was used to inhibit post-transcriptional gene silencing and to allow other constructs to be expressed (Roth et al., 2004).

• L.148-150 P.5: How are overexpression artifacts determined? Maybe add a timecourse of expression from 1-4 days to illustrate the issue.

Overexpression artifacts can be proteins accumulating into upstream compartments (ER in the case of Golgi targeting for example). We routinely image samples 3 days after infiltration when we are interested in determining where a protein localises. We have added the following sentence in the text to clarify why: The time point of 3 dpi was selected because in our hands there is no detectable protein expression 1 dpi, and at 5 dpi expression levels may be too high and proteins may start to accumulate in other compartments.

• L. 176-183: were notch filters applied in the Leica SP8? If yes, the wavelengths needs to be added.

Yes, in most cases. We have now added this info in the material and methods section.

• In general, there is a very confusing misorder in the figure references. Moreover, Figure 1 is a table while there are references to figure 1B as the first reference.

Figures were cited in their order of appearance in the text (first call for Figure 1 and Figure 2 at line 186 in the previous version of the manuscript, and Figure 2B was called at line 194 in the previous manuscript, etc). There was no call for Figure 1B in the manuscript as there was no Figure 1B in the previous manuscript. The confusion may stem from the fact that Figure 1 looked like a table. Our manuscript was transferred directly from another journal which stipulated that a table using colours must be called a figure. The guidelines of Plant Direct are different and we are now able to call this table, Table 1 (formerly Figure 1). We hope that this will alleviate the confusion. Our manuscript now contains 7 figures and 3 tables.

Reviewer #2:

Review - I see the light! Fluorescent proteins suitable ffor cell wall/apoplast targeting. (pH resilience of apoplastic fluorescent proteins)

The manuscript by Stoddard and Rolland investigates the fluorescence stability of a number of fluorescent proteins (FPs) under acidic pH conditions with the purpose of finding suitable FPs for investigating apoplastic protein dynamics and activity.

General comments

Overall, the manuscript is well written outlining the purpose of the research and the findings clearly. The research has meaning and impact for those working on the apoplast, cell walls and secretory systems where the pH of the compartments change. There are a few grammatical errors that should be fixed throughout the text but these do not affect the overall message.

Thank you for your positive feedback about our manuscript. If Reviewer 2 could point out the grammatical errors which we did not spot, we will gladly fix them!

The work was done using the transient expression system of Nicotiana benthamiana looking at mesophyll and epidermal cell layers. The conclusion that mTurquoise2 is the better FP to use rather than mCherry is partly based on the autofluorescence of the sample, which I assume is the chloroplast autofluorescence. While this can be a problem, there is also the autofluorescence in most plants and tissues in the blue and cyan wavelengths from cell wall components such as ferulic acids, lignins etc and in the vacuole in response to pathogen attack. I would recommend that the conclusion is adjusted and broadened to take this into consideration.

This is a good point. With the inclusion of 3 new FPs and fluorescence quantification, we have restructured our conclusion (see "General comments to the Editors and Reviewers" above). We have added a Summary and Conclusion section at the end of the results section to briefly go over the three groups. We have also added a new section to our discussion to specifically discuss the issue of fluorescence and other aspects to consider when choosing the best FP for a given experiment.

Following on from the assessment of the fluorophores, there is no comparative intensity information of the fluorescence in vivo. While the fluorescence changes are visible, simple analysis of the intensity of the fluorescence in the cytoplasm and the apoplast would show the fluorescence semi-quantitatively. While this is not a direct comparison between the constructs, it supports the premise of the paper.

This is a fantastic suggestion, which was also made by reviewer 1.

Quantifying the fluorescence in the experiments where we expressed the FPs in the cytosol and the apoplast (now Figure 3 and Figure 4) would not be meaningful because they are done in different cells and even different compartments. These experiments are meant as a rough indication as to whether or not the same FP can be detected in these 2 compartments using the same confocal settings.

However, quantifying the fluorescence in our pH series is a very valuable experiment because it can be done in the same group of cells across the pH series and allow for relative comparisons to be made. Three pH series were quantified for each one of the 9 FPs tested (a total of 27 pH series - Citrine was not tested because we could not detect it in the apoplast). This quantification is presented in a new figure (Figure 7) a new table (Table 2). We have also added a new paragraph to the Mat and Met section to explain how we quantified the fluorescence. In short, to account for slight tissue rotation induced by the repeated replacement of buffered solutions, the cumulative fluorescence value (called RawIntDen in Image J) of a disc of 512 µm in diameter was measured in each image. The chlorophyll channel (not shown) was used to ensure that discs were located over the same group of cells across a pH series. Background fluorescence (as measured in a disc of 512 µm in diameter expressing P19, placed in 50mM HEPES pH 8, and imaged with identical settings) was deducted from each measurement in a given pH series. Fluorescence intensities were then normalised to the fluorescence measured at pH 8 (arbitrarily set at 100%).

The results of this quantification show that fluorescent proteins fall into two categories:
- mTurquoise2, Gamillus, mNeonGreen, TagRFP, mCherry retained ~>50% of their pH 8 fluorescence when placed in a pH 4 buffer. This group can further be split in two, depending on whether or not they aggregated in the cytoplasm (see general comment above).
- sfGFP, pH-tdGFP, mEYFP, mCitrine retained <10% of their pH 8 fluorescence when placed in a pH 4 buffer. These proteins are considered unsuited to the apoplast.

We are extremely grateful to both Reviewers for this suggestion as we believe it has made our manuscript much stronger!

Specific comments

Page 4, line 122 - The genes RPP3A and AT5G11420 are mentioned here in the Materials and Methods but what the genes are isn't listed until Page 7, lines 218 and 220 respectively in the results. This information should be moved to the final paragraph of the introduction, e.g., Page 4, line 103, to prevent readers searching for the information.

Good point – we have followed Reviewer 2's comment and moved the description of the genes where suggested.

Page 5, line 155 - how big are the leaf discs that are imaged? Does the size impact the viability of the cells for live cell imaging? Were the leaf discs infiltrated for plasmolysis experiments post-excision and if so did this cause damage to the cells being imaged?

In this study, the discs were all of the same size (2.2 cm, which is quite large) and were imaged within 15-20 minutes from being harvested. Images were always taken away from the tissue damaged by the syringe nose and away from the edge of the disc to ensure that we were looking at undamaged cells. Given the size of the discs used here, the area of undamaged tissue available corresponds to the vast majority of the disc. Regarding plasmolysis experiments, the discs were infiltrated immediately before excision. Cells do get stressed by plasmolysis but we did not observe any other kind of damage.
